# Assessment of COVID-19-related right ventricular morphological and functional alterations and evaluation of their impact on the course of the disease

**DOI:** 10.1186/s13613-024-01278-2

**Published:** 2024-03-26

**Authors:** Michael Dandel

**Affiliations:** German Centre for Cardiovascular Research, 13347 Berlin, Germany

Dear Editor,

I read with great interest the article by Jozwiak et al. published in *Annals of Intensive Care* [1]. Particularly important in that study, which investigated the incidence and severity of RV injury in critically ill patients with COVID-19, is the use of a standardized not clinically driven transthoracic echocardiographic (TTE) follow-up during the intensive care unit (ICU) stay, at predetermined times. That approach was indeed new because during the COVID-19 pandemic, there was a general tendency to follow the initial recommendation to avoid “unnecessary” echocardiographic examinations in order to reduce transmission of the virus and protect the medical staff [2], although the praxis demonstrates that, without any echocardiographic examination, it cannot be established whether a TTE is indeed unnecessary in a given patient who requires intensive care. In this regard, as well as in view of the several important details provided by the study, the authors deserve special appreciation. Nevertheless, few aspects of the study which have received less attention are worth considering.

The probably most important limitation which was also mentioned by the authors, but not described in more detail, is the omission of specific assessment of tricuspid regurgitation (TR) regarding its severity and its potential impact on RV anatomical and functional parameter measurements. It is proven that, correspondingly to the increased blood volume leaving the RV in systole, TR can induce overestimations of RV contractile function by facilitating both the RV free wall longitudinal motion which will increase the tricuspid annular plane systolic excursion and its velocity (TAPSE and TAPSV, respectively), and the transversal (inward) RV wall motion which additionally also contributes to the increase in the RV fractional area change (FAC) [3]. Given that TR was present in 60% of the TTE examinations included into the study by Jozwiak et al. [1], in retrospect, the initial decision to forego the potential benefits of a detailed TR severity grading appears less appropriate. The possible impact of relevant TR could, for example, contribute to the otherwise hardly explicable similar TAPSE and RV FAC values measured in patients without RV injury and those with relevant RV dilation, which were even slightly higher in those with RV dilation (i.e. 22 mm vs. 23 mm for TAPSE, and 47% vs. 49% for FAC [1]). The Fig. 1A presented by Jozwiak et al. in their article, which illustrates an example of isolated RV dilation in a patient with normal RV FAC (≥ 35%) clearly indicates (indirectly) the presence of a relevant TR. Thus, comparing in that patient the right atrial (RA) size at end-diastole, with that at the end-systole, an unequivocal RA dilation during the RV systole becomes clearly apparent. This in turn indicates the presence of a relevant TR which can contribute to the preservation of a normal RV FAC, even in the presence of a reduced forward stroke volume.

An important finding provided by Jozwiak et al. (Table S2) [1] which deserves special consideration is related to the high ICU and overall mortality rates in the 18 patients with initially only RV dilation (i.e. 44% and 56%, respectively) which were considerably higher than those revealed by the 28 patients with initially only signs for RV dysfunction (TAPSE and FAC reduction, without RV dilation) and the small group (5 patients) with both RV dilation and dysfunction (i.e. 18% and 25%, respectively, and 0% and 20%, respectively). This appears at first sight not in line with the fact that RV dilation alone is not a direct cause of death, and also not with the highly plausible finding in the study, that only combined RV dilation and dysfunction were independently associated with a significant increase in mortality risk. However, given the high incidence of extensive pulmonary thrombotic microangiopathy associated with severe ventilation-perfusion mismatch, increased resistance to pulmonary blood flow, and frequent afterload-induced acute RV failure (which were also identified as distinct features of severe COVID-19-related ARDS) [3–5], the emergence and progression of RV dilation which was consistently reported during the pandemic as an important early sign for potential future life-threatening deteriorations of pulmonary circulation associated with RV pressure overloading-induced RV failure, needs particular consideration [3, 5]. In patients without pre-existing cardiac diseases, a reduction of TAPSE and/or FAC without RV dilation in the presence of a RV pressure overloading indicates the existence of RV systolic dysfunction which is still able to prevent RV-pulmonary arterial uncoupling associated with the development of RV failure. The high mortality rates found by Jozwiak et al. in patients with initially only RV dilation could indicate that early dilation, before emergence of relevant RV systolic dysfunction, may reflect a lower RV adaptability of to acute pressure overloading. Thus isolated progressive RV dilation can be an important early prognostic sign, able to facilitate the timely identification of high-risk patients. Therefore, monitoring of the RV size and both emergence and progression of TR by TTE deserve particular attention in COVID-19-related ARDS.

## Data Availability

Not applicable.

## References

[1] Jozwiak M, Dupuis C, Denormandie P (2024). Right ventricular injury in critically ill patients with COVID-19: a descriptive study with standardized echocardiographic follow-up. Ann Intensive Care.

[2] Kirkpatrick JN, Mitchell C, Taub C (2020). ASE Statement on Protection of patients and Echocardiography Service Providers during the 2019 Novel Coronavirus Outbreak: endorsed by the American College of Cardiology. J Am Soc Echocardiogr.

[3] Dandel M (2022). Heart-lung interactions in COVID-19: prognostic impact and usefulness of bedside echocardiography for monitoring of the right ventricle involvement. Heart Fail Rev.

[4] Fox SE, Akmatbekov A, Harbert JL (2020). Pulmonary and cardiac pathology in African American patients with COVID-19: an autopsy series from New Orleans. Lancet Respir Med.

[5] Dandel M (2021). Pathophysiology of COVID-19-associated acute respiratory distress syndrome. Lancet Respir Med.

